# Supplementary material for: Differential voltage-dependent modulation of the ACh-gated K+ current by adenosine and acetylcholine
Source: PLoS One. 2022 Jan 14;17(1):e0261960. doi: 10.1371/journal.pone.0261960 (PMC8759768; doi:10.1371/journal.pone.0261960)
Supplement: S1 Table — (DOCX) [file pone.0261960.s001.docx]

**S1 Table.** **Individual parameters obtained from the fits of the C-R relationships for Ado**

|  | **Voltage** | **Cell#1** | **Cell#2** | **Cell#3** | **Cell#4** | **Cell#5** | **Cell#6** | **Cell#7** | **Cell#8** | **Cell#9** | **Mean ± SEM** | ***P***^a^ |
| --- | --- | --- | --- | --- | --- | --- | --- | --- | --- | --- | --- | --- |
| **E_max_** | -100 mV | 0.97 | 1.01 | 0.98 | 0.97 | 1.01 | 1.03 | 0.99 | 1.01 | 0.99 | 1.00 ± 0.01 | 0.16 |
|  | +30 mV | 0.94 | 0.99 | 0.98 | 0.96 | 1.01 | 1.01 | 1.01 | 0.92 | 0.99 | 0.98 ± 0.01 |  |
| **n_H_** | -100 mV | 1.01 | 1.15 | 1.19 | 1.09 | 0.73 | 0.66 | 0.93 | 1.57 | 0.90 | 1.02 ± 0.09 | 0.98 |
|  | +30 mV | 1.09 | 1.11 | 1.37 | 1.29 | 0.68 | 0.63 | 0.59 | 1.65 | 0.83 | 1.03 ± 0.12 |  |
| **pEC_50_** | -100 mV | 6.48 | 6.53 | 6.71 | 6.44 | 6.49 | 6.37 | 6.69 | 7.10 | 6.76 | 6.62 ± 0.07 | 0.03^*^ |
|  | +30 mV | 6.42 | 6.31 | 6.48 | 6.35 | 6.37 | 6.41 | 6.71 | 7.09 | 6.69 | 6.54 ± 0.08 |  |

^a^*P* = probability value; *, P < 0.05. As indicated in Methods, the paired *t* test was used for analyzing statistical significance, except for E_max_ for which The Wilcoxon signed rank test was applied.
