# Supplementary material for: Differential voltage-dependent modulation of the ACh-gated K+ current by adenosine and acetylcholine
Source: PLoS One. 2022 Jan 14;17(1):e0261960. doi: 10.1371/journal.pone.0261960 (PMC8759768; doi:10.1371/journal.pone.0261960)
Supplement: S2 Table — (DOCX) [file pone.0261960.s002.docx]

**S2 Table.** **Individual parameters obtained from the fits of the C-R relationships for ACh**

|  | **Voltage** | **Cell#1** | **Cell#2** | **Cell#3** | **Cell#4** | **Cell#5** | **Cell#6** | **Mean ± SEM** | ***P***^a^ |
| --- | --- | --- | --- | --- | --- | --- | --- | --- | --- |
| **E_max_** | -100 mV | 0.97 | 0.95 | 0.96 | 1.04 | 0.98 | 0.96 | 0.98 ± 0.01 | 0.14 |
|  | +30 mV | 0.99 | 1.03 | 1.04 | 1.00 | 1.03 | 0.97 | 1.01 ± 0.01 |  |
| **n_H_** | -100 mV | 1.19 | 1.25 | 1.25 | 0.97 | 1.25 | 1.49 | 1.23 ± 0.07 | 0.13 |
|  | +30 mV | 1.29 | 0.97 | 1.05 | 1.11 | 0.81 | 1.02 | 1.04 ± 0.06 |  |
| **pEC_50_** | -100 mV | 6.41 | 6.41 | 6.28 | 5.92 | 6.40 | 6.64 | 6.34 ± 0.10 | 0.01* |
|  | +30 mV | 6.20 | 5.77 | 5.79 | 5.92 | 5.81 | 5.91 | 5.90 ± 0.06 |  |

^a^*P* = probability value; *, P < 0.05. As indicated in Methods, the paired *t* test was used for analyzing the statistical significance.
