# Supplementary material for: Differential voltage-dependent modulation of the ACh-gated K+ current by adenosine and acetylcholine
Source: PLoS One. 2022 Jan 14;17(1):e0261960. doi: 10.1371/journal.pone.0261960 (PMC8759768; doi:10.1371/journal.pone.0261960)
Supplement: Attachment — Submitted filename: Responses to reviewers.docx [file pone.0261960.s003.docx]

We sincerely thank the editor and reviewers for the positive constructivism on our study and the suggestions for improvement of our manuscript. Below we paraphrase your specific comments and provide a detailed response in blue font. Changes in the manuscript are denoted in red font.

Note: The line and page numbers indicated here are designated for the Revised Manuscript with Track Changes.

**Responses to Editor's comments:**

In addressing the specific points of the reviewers, we ask that 1) a more rigorous statistical analysis be performed and described in the Methods and/or figure legends corresponding to the analyses

R = We have added a little bit more information in Methods about this issue and complemented in Results and Figure legends the tests used. Moreover, in order to clarify the statistical analysis (especially in Fig. 1), we added Suppl. Table 1 with the individual parameters (E_max_, n_H_, and pEC_50_) of the C-R relationships for adenosine, which shows paired data (-100 mV versus +30 mV) for each myocyte recorded.

and 2) a tract be added to the Discussion which clearly and adequately distinguishes your current findings from earlier work published by your group.

R = Several lines were inserted in the Introduction (line 57, page 3 and lines 73-75, page 4) and the Discussion (lines 258-261, page 12) to better distinguish our findings herein from those previous ones.

Journal Requirements:

When submitting your revision, we need you to address these additional requirements.

1. Please ensure that your manuscript meets PLOS ONE's style requirements, including those for file naming. The PLOS ONE style templates can be found at 
<https://journals.plos.org/plosone/s/file?id=wjVg/PLOSOne_formatting_sample_main_body.pdf> and 
<https://journals.plos.org/plosone/s/file?id=ba62/PLOSOne_formatting_sample_title_authors_affiliations.pdf>

R = Done.

2. Thank you for stating the following in the Acknowledgments Section of your manuscript: 
[Doctoral Fellowships were awarded to A.L.L-S., R.Z-C., and P.D.S-F. (#286520, 587036, and 231965, respectively) from CONACYT, Mexico. The authors wish to thank Miguel Angel Flores-Virgen for technical assistance.]
We note that you have provided funding information that is not currently declared in your Funding Statement. However, funding information should not appear in the Acknowledgments section or other areas of your manuscript. We will only publish funding information present in the Funding Statement section of the online submission form. 
Please remove any funding-related text from the manuscript and let us know how you would like to update your Funding Statement. Currently, your Funding Statement reads as follows: 
 [This work was supported by SEP-CONACYT, Mexico. Grant No. CB-2011-01-167109 (to E.G.M-G.), and CB-2013-01-220742 (to R.A.N-P). The funders had no role in study design, data collection and analysis, decision to publish, or preparation of the manuscript.]

Please include your amended statements within your cover letter; we will change the online submission form on your behalf.

R = Done.

**Responses to Reviewers' comments:**

**Reviewer #1:**

In this paper, Lopez-Serrano examined the modulation of GIRK currents by Adenosine and ACh in guinea pig cardiac myocytes. The authors have shown in previous publications how ACh can modulate GIRK currents in a voltage-sensitive manner. In the current study, they compared Adenosine with ACh effects. Their results confirm the ACh effects, while Adenosine does not appear to modulate GIRK channels in a similar manner. Overall, the paper is well written and logically presented. However, the findings presented with ACh are not novel. The authors have shown similar findings previously, albeit in cat cardiac myocytes. They have not presented a clear justification of why they are replicating previous work. There is no insight provided (nor learned) on the mechanism by which adenosine may be different from ACh even though they use the same Gai/o G proteins.

R = You’re right, ACh results were previously shown in feline cardiac cells. Although, when we wanted to explore the effects of membrane potential on the A_1_R-G_i/o_-*I*_KACh_ signaling pathway, to our surprise, it was not functional (at least in our hands) in this species. That’s why, to validate our Ado-A_1_R findings in guinea-pig cardiomyocytes, we considered important to know what was going on with the other concurrent signaling pathway (M_2_R-G_i/o_-*I*_KACh_) in this same species. Therefore, by using this cellular model we now know that: 1) voltage has no influence (or it is very weak) on the A_1_R-G_i/o_-*I*_KACh_ signaling pathway when it is activated by its physiological agonist Ado; 2) the ACh results are important because, as commented by reviewer #2, they act as a positive control for Ado results; and 3) here we show for the first time that *I*_KACh_ is differentially modulated by voltage by being coupled to both A_1_R and M_2_R. These results envisage that the fine-tuning of the voltage-dependent cellular responses through a given same effector is still much more dynamic and complex when it is coupled to two or more GPCRs. We have added several lines in the Introduction (line 57, page 3 and lines 73-75, page 4) and the Discussion (lines 258-261, page 12) to better singularize our findings herein from those in our previous works.

Regarding the mechanism, you’re right, thanks for the suggestion, we have included some argumentation in the Discussion (lines 248-255, pages 11-12) trying to explain why ACh and Ado could have differential voltage-dependent effects on their concurrent downstream effectors.

In regards to Figure 1B, the authors state that the Adenosine C-R obtained at -100 mV was significantly different (P<0.03) than the C-R obtained at +30 mV. This does not seem to be the case and the authors do not state what statistical test they employed to arrive to this conclusion. They must provide the test to the reader, otherwise it is not convincing.

R = Apart from the general description in Methods, we now mentioned the tests used in the text of Results and Figure legends. Thanks to this observation, we decided it was important to show the individual parameters (E_max_, n_H_, and pEC_50_) of the C-R relationships for adenosine as a Suppl. Table 1, where it can be observed that, despite the tiny difference of the means (especially for pEC_50_), it is easier to understand the statistical significance of the difference by observing the paired data (-100 mV versus +30 mV) in each of the recorded cells.

**Reviewer #2:**

The manuscript "Differential voltage-dependent modulation of the Ash-gated K current by adenosine and acetylcholine," by Lopez-Serrano et al., describes a study aimed at determining whether membrane voltage alters adenosine receptor function. Previous work had shown the muscarinic receptors can exhibit altered function with strong changes in membrane potential, including changes in agonist affinity and/or binding. Here the authors examined adenosine receptors in cardiac myocytes to test whether they exhibited similar voltage dependent changes. They found that apparent agonist affinity (measured using I-KAch as an assay for receptor activity and current deactivation as a proxy for ligand-receptor unbinding) appeared unaltered. Authors also reported no change in I-KAch relaxation with membrane potential. By contrast, authors show that similar parameters were altered when currents were activated by acetylcholine, as previously reported, thus acting as a positive control. Ultimately the data provided were compelling that adenosine receptors do not show voltage dependence.

Minor issues:
In some figures (figs 2&5), authors display scatter plots illustrating raw data points, but in most figures they do not. Although it may clutter the figures, showing all of the raw data points would be preferable.

R = To be consistent in all figures, we have updated Figs. 1, 3, 4, and 6 depicting the individual raw data points, as requested.

Fig. 4 shows dose-response curves for Ach responses. Curves do not seem to reach saturation, so it's unclear how accurate the EC50 fits are, nor whether changes in efficacy were evident (since data are only displayed as normalized to max).

R = Good observations. That’s why we realized that it would be illustrative to include the Suppl. Table 2 with the individual parameters of the C-R curves for ACh, where it can be observed that the estimated E_max_ values were virtually equal to 1 (0.98 ± 0.01 at -100 mV and 1.01 ± 0.01 at +30 mV), and hence these data clearly show that the accuracy of the EC_50_ fits is adequate by reaching saturation of the response at both voltages studied.

Regarding the possible change in efficacy with voltage, we have to bear in mind that

due to the nature of K_ACh_ channels (a K^+^ conductance that exhibits characteristic inward rectification, i.e., a voltage-dependent block by intracellular Mg^2+^ and polyamines), the magnitude of the current *I*_KACh_ (either the absolute (pA) or the density (pA/pF)) at a given membrane potential depends on both the driving force and the aforementioned rectification. Therefore, in this type of experiments (functional) it is not possible to assess the efficacy of physiological or other full agonists using E_max_ with the current magnitude. Instead, it must be used a saturating concentration at both the negative and positive voltages, which will indicate the maximum activation of the signaling pathway. With this kind of experiments, the change in efficacy can be studied for partial agonists when compared with a saturating concentration of the physiological agonist, as we have previously shown in this signaling pathway for pilocarpine, choline, and tetramethylammonium (refs. 9, 15, and 16 in the manuscript).

**Reviewer #3:**

This is an interesting report on how the activation of adenosine and acetylcholine receptors can modulate the current of K+ activated by acetylcholine (IKACh) in cardiac myocytes. In this work, the authors find a voltage-dependent differential modulation of IKACh by Ado and ACh. In the first case, the influence is null, while the second showed an apparent voltage-dependent effect on IKACh and induced a property known as "relaxation" of the current. Even though both agonists activate the same signaling pathway, the authors argue that both GPCRs show a different voltage sensitivity that modifies their affinity for the ligand that could help explain their differential effects on IKACh. In this reviewer's opinion, it is an interesting work, correctly planned and executed, clearly written in which the data support the authors' conclusions. However, I have a few considerations for the authors:
1) In the description of Figure 1B, the authors mention that the results of the analysis of the IKACh data obtained at -100 and +30 mV after applying Ado at different concentrations are statistically different. However, the data in the graph shows that this may not be the case. The data is very similar. This statement by the authors is actually contrary to their hypothesis (as shown by the results of Figs. 1-3) that Ado does not modify the properties of IKACh. For this reason, conducting a more rigorous and complete statistical analysis would strengthen the paper. This analysis should consider information on how the statistical analysis was conducted in Figs 1B and 4B (whether the t-test is the best option; and indicate the exact P values).

R = Please, see our response to the editor and reviewer #1 about this issue. Briefly, we explained a little bit more in Methods and mentioned in Results and Figure legends the test used next to the exact P values calculated. Importantly, in this revised version we are including the Suppl. Table 1 and 2 to display the individual parameters (E_max_, n_H_, and pEC_50_) of the C-R relationships for Ado and ACh, where the paired data (-100 mV versus +30 mV) for each myocyte can be seen, and thus better appreciate the statistical significance of the differences.

2) On the other hand, this reviewer would not recommend using scatter plots with means ± SD to represent experimental data (specifically in Figs. 2B and 5B). Instead, box-and-whisker plots that provide more information to summarize data should be used. The authors should replace these plots with box-and-whisker plots, with the box showing the median and the 25th and 75th quartiles and the whisker representing the 5th and 95th percentile. In addition, it would be nice if data points could be added to the plot superimposed on the box-and-whisker graphs. It sounds complicated, but fortunately, there are powerful statistical software packages available that can give these results directly.

R = We respectfully disagree with this observation. The median and quartiles values (displayed in box-and whisker graphs) are recommended for non-symmetric distributions (Krzywinski & Altman, 2014; *Nat Methods* 11: 119-120), whereas symmetric distributions are better described using the mean and SD (or SEM) (Drummond & Tom, 2011; *J Physiol* 589: 5007-5011), which was our case as checked with the normality Shapiro-Wilk test or even by eye in Figs. 2B and 5B.

3) Perhaps it would be worthwhile for the authors to add a paragraph to the discussion section regarding the characteristics, if known, in the molecular machinery that causes Ach receptors to have a higher affinity for the ligand at different voltages.

R = Thanks for the suggestion. In the Discussion (lines 248-255, pages 11-12), we have extended the argumentation not only about the possible mechanism for ACh but also trying to discern the differential voltage-dependent effects of Ado and ACh on their coexisting downstream effectors.
