# Supplementary material for: Differential voltage-dependent modulation of the ACh-gated K+ current by adenosine and acetylcholine
Source: PLoS One. 2022 Jan 14;17(1):e0261960. doi: 10.1371/journal.pone.0261960 (PMC8759768; doi:10.1371/journal.pone.0261960)
Supplement: Attachment — Submitted filename: Responses to reviewers R2.docx [file pone.0261960.s004.docx]

Note: The line numbers designated here are referred for the Revised Manuscript with Track Changes.

**Responses to Editor's comments:**

Given the marginal significance of the p value indicated for Ado in experiments shown in Fig. 1, we ask that the persistent points of Reviewer 1 be addressed as requested and/or that the implications of a borderline p value in this particular case be discussed.

R = Certainly, we are conscious of the borderline p value obtained in the C-R curves that appear to be very close with each other. Although, this can be explained by the power of statistical analysis with paired data and, as known, the statistical significance will be greater when increasing the number of experiments. Anyway, we recognize that this is a *very weak* voltage-dependent behavior that is not mirrored in other less sensitive functional assays, such as the deactivation kinetics and with voltage step protocols (lines 247-251). In the manuscript, we are aware of this situation by stating that there is a *null or very weak* influence of voltage on *I*_KACh_ when activated by Ado through A_1_R (lines 153-154, 189-190, and 219-220).

Furthermore, thanks to your suggestion, in the Discussion we rephrased some lines trying to better clarify that, although Ado activates A_1_R-*I*_KACh_ with very slight voltage dependence, our findings implicate that A_1_R is voltage sensitive, which in turn suggests that other adenosine agonists could induce diverse voltage-dependent patterns, as has been found in other GPCRs (lines 251-256).

Please also tend to the minor change to Fig. 1B.

R = Done. In this R2 version, we have removed the individual raw data points to only show the mean values in Fig. 1 (Ado C-R curves), as requested by reviewer #1. However, as you may remember, reviewer #2 previously requested us to update Figs. 1, 3, 4, and 6 by showing both the means (±SEM) and individual raw data points to be consistent with Figs. 2 and 4, and so we did in the R1 version. At this point, we think that it is inconsistent that Figs. 2-6 display individual raw data points while Fig. 1 does not. Therefore, we would like to ask you to decide whether we should display both the means and individual raw data points in all figures or to remove the individual raw data points in Figs. 1, 3, 4, and 6 (not in Figs. 2 and 5 where it is very important to depict the correlation of paired data). Any of these alternatives if ok for us.

**Responses to Reviewer' comments:**

**Reviewer #1:**

In the resubmission, the authors have clarified the reason for replicating previous studies. Additionally, it was requested that the authors indicate the statistics used to conclude that the C-R curves in Figure 1B were "displaying a slight but statistically significant (P=0.03; paired t-test) greater potency at the negative potential". In the resubmission, the authors have provided 2 additional tables with the individual parameters generated by the fits for each cell. Why don't the authors run a test to determine whether the fit for one curve is different from the other curve?

R = We don’t exactly understand about whether the fit for one curve is statistical different from the fit of the other curve. Just in case, we calculated the goodness of the individual fits (R^2^) for every cell at both studied voltages, resulting 0.996 ± 0.001 (-100 mV) and 0.991 ± 0.005 (+30 mV), which were statistical similar (p = 0.50; Wilcoxon signed rank test); consequently, the statistical significance observed for the pEC_50_ was not result of the goodness of the individual fits, these were good enough at both potentials. In other words, the fits were not determinant for the significant result of the EC_50_/pEC_50_. We are sure that the significant value of pEC_50_ was due to the power of statistical analysis by using only paired data. That’s the importance of displaying Table S1 with the individual parameters for each of the cell, where it can be observed that despite of the tiny difference the direction of the change was prevalent (higher pEC_50_ at -100 mV) Moreover, as known, if there was statistical significance with a n = 9, it will certainly be greater when increasing the number of experiments.

Also, why did the authors exclude the EC50 values in both Tables 1 and 2? Is there a reason why the authors chose to compare the difference in pEC50 for each cell instead of the EC50? Did they observe the same statistical differences when comparing EC50 values?

R = In publications, it is usual that measurement of *potency* in C-R (or D-R) curves is denoted in the form of EC_50_. Although, a formal analysis of the estimation of potency (not to mention the special case herein with Ado) requires that the parameters are expressed as p-values (i.e., pEC_50_) because such estimations are done with the p-scale (-log of the value) and, as they are log normally distributed, only these values should be used for statistical analysis (T.P. Kenakin, 2009; *A Pharmacology Primer: Theory, Applications, and Methods*; 3^rd^ ed.; pgs. 17-19). The use of this formal practice can be checked in many publications in well-respected Journals, for example: Giakomidi D et al., 2021; *PLoS ONE* 16: e0250011; Perdona E et al., 2019; *Neurosci Lett* 711: 134456; Schrage R et al., 2013; *Brit J Pharmacol* 169: 357-370; Sahlholm K et al., 2011; *Neuropharmacol* 61: 937-949; Liapakis G et al., 2004; *Mol Pharmacol* 65: 1181-1190; etc.

Finally, the authors compared the differences of the pEC50 means, and those were significantly different. But the pEC50 values were not compared. So it is incorrect to state that there was a statistically significant greater potency.

R = Fig. 1B was constructed with the mean values of the different concentrations for all assayed cells, while the fits are shown for illustrative purposes. For the statistical analysis, the parameters (E_max_, n_H_, and pEC_50_) of the C-R relationships were estimated using the Hill equation with the individual measurements (normalized currents) from each myocyte, and thereby the pEC_50_ values mentioned in the text (6.54 ± 0.08 at +30 mV and 6.62 ± 0.07 at -100 mV) represent the means of these individual parameters from the nine myocytes. These same data were used for the statistical analysis (normality test followed by the paired *t* test), resulting a significant value of p = 0.03. As commented above, this was due to the power of statistical analysis by using only paired data and, as observed in Table S1, despite of the tiny difference, the direction of the change was dominant in the cells evaluated (higher pEC_50_ at -100 mV).

Anyway, in the manuscript we are aware that this is a *very weak* voltage-dependent behavior that is not reflected in the other less sensitive functional assays (lines 247-251), and thus we always refer that there is a *null or very weak* influence of voltage on *I*_KACh_ when activated by Ado through A_1_R (lines 153-154, 189-190, and 219-220). Additionally, as suggested by the editor, in the Discussion we rephrased some lines trying to better clarify the implications of a borderline p value in this particular case (lines 251-256).

Minor: For figure 1B, the authors should remove the individual raw data points and just show the mean values.

R = Done. Even though, please see our response to the editor about this point.
